# Rationale for Selective Muscarinic Receptor Agonists as Candidates for the Management of Cognitive and Neuropsychiatric Symptoms in Alzheimer's Disease

**DOI:** 10.1002/alz70855_105646

**Published:** 2025-12-24

**Authors:** Ronald Marcus, Scott Vuocolo, Ian Ramsey, Carolyn Watson, Minsu Kang

**Affiliations:** ^1^ Bristol Myers Squibb, Princeton, NJ, USA

## Abstract

**Background:**

Cognitive impairment and neuropsychiatric symptoms, including psychosis, are fundamental features of Alzheimer's disease (AD) and related dementias. Currently treatments for these symptoms have efficacy, safety, and tolerability limitations; new treatments with novel mechanisms are needed. Previous research has shown that the M_1_/M_4_ preferring muscarinic receptor agonist xanomeline improved cognition and psychosis in people living with AD and provided support for investigating this class of agent for these symptoms in AD.

**Methods:**

A literature review was performed to identify preclinical data on muscarinic receptor regulation of neural networks implicated in psychosis and cognition. The evidence gathered was used to develop hypotheses about the potential role of muscarinic receptors in the regulation of psychosis and cognitive pathways in AD.

**Results:**

Muscarinic receptors are highly expressed in brain areas related to psychosis (striatum, prefrontal cortex [PFC]) and learning and memory (frontal cortex, hippocampus). One hypothesis for the observed antipsychotic efficacy of muscarinic receptor agonists posits that activation of presynaptic M_4_ muscarinic autoreceptors on acetylcholine‐producing neurons projecting from the laterodorsal tegmentum to the medial ventral tegmental area (VTA) inhibits further acetylcholine release and thereby lowers excitation of dopamine circuits originating in the VTA. A second hypothesis suggests that activation of M_1_ muscarinic receptors on GABA interneurons that terminate on cortical glutamatergic neurons dampens cortical excitatory glutamatergic pyramidal cells terminating in the VTA. Regarding cognition, activation of M_4_ muscarinic receptors in excitatory pyramidal cells in the CA3 area of the hippocampus will inhibit glutamate release, which lowers excitatory drive and inhibits pyramidal neurons in the CA1 area. Finally, activation of M_1_ muscarinic receptors on GABA interneurons in the PFC results in increased GABA release, which may normalize the excitation/inhibition balance in cortical areas associated with cognition.

**Conclusions:**

M_1_ and M_4_ muscarinic receptors are thought to impact psychosis and cognition by modulating the level of neurotransmitters via distinct pathways. Importantly, the location of muscarinic receptors in certain areas of the brain dictates specific neurotransmitter responses in those areas, as opposed to widespread changes throughout. Overall, muscarinic receptor agonists may represent a new therapeutic class for the treatment of cognitive and neuropsychiatric symptoms of AD.

